# Adverse maternal outcomes associated with fetal macrosomia: what are the risk factors beyond birthweight?

**DOI:** 10.1186/1471-2393-13-90

**Published:** 2013-04-08

**Authors:** Florent Fuchs, Jean Bouyer, Patrick Rozenberg, Marie-Victoire Senat

**Affiliations:** 1 Department of Obstetrics and Gynecology, Hôpital Béclère-Bicêtre, Assistance Publique Hôpitaux de Paris (APHP), Le Kremlin-Bicêtre, France; 2 Inserm, CESP Centre for Research in Epidemiology and Population Health, U1018, Reproduction and Child Development, Villejuif F-94807, France; 3 Univ Paris-Sud, UMRS 1018, Villejuif F-94807, France; 4 Department of Obstetrics, Hôpital Poissy-Saint Germain, Versailles-St Quentin University, Poissy, France; 5 Hôpital Bicêtre, Service de Gynécologie-Obstétrique, 78 rue du Général Leclerc, Le Kremlin-Bicêtre cedex 94275, France

**Keywords:** Macrosomia, Maternal outcomes, Postpartum hemorrhage, Perineal tears

## Abstract

**Background:**

To identify risk factors, beyond fetal weight, associated with adverse maternal outcomes in delivering infants with a birthweight of 4000 g or greater, and to quantify their role in maternal complications.

**Methods:**

All women (n = 1564) with singleton pregnancies who attempted vaginal delivery and delivered infants weighing at least 4000 g, in two French tertiary care centers from 2005 to 2008, were included in our study. The studied outcome was maternal complications defined as composite item including the occurrence of a third- or fourth-degree perineal laceration, or the occurrence of severe postpartum hemorrhage requiring the use of prostaglandins, uterine artery embolization, internal iliac artery ligation or haemostatic hysterectomy, or the occurrence of blood transfusion. Univariate analysis, multivariable logistic regression and estimation of attributable risk were used.

**Results:**

Maternal complications were increased in Asian women (adjusted odds ratio [aOR], 3.1; 95% confidence interval [CI], 1.1–9.3, Attributable risk (AR): 3%), in prolonged labor (aOR = 1.9 [95% CI; 1.1–3.4], AR = 12%) and in cesarean delivery during labor (aOR = 2.2 [95% CI; 1.3–3.9], AR = 17%). Delivering infants with a birthweight > 4500 g also increased the occurrence of maternal complications (aOR = 2.7 [95% CI; 1.4–5.1]) but with an attributable risk of only 10%. Multiparous women with a previous delivery of a macrosomic infant were at lower risk of maternal complications (aOR = 0.5 [95% CI; 0.2–0.9]).

**Conclusion:**

In women delivering infants with a birthweight of 4000 g or greater, some maternal characteristics as well as labor parameters may worsen maternal outcome beyond the influence of increased fetal weight.

## Background

A consistent increase in the mean birthweight and in the proportion of fetal macrosomia, defined as a birthweight greater than 4000 g, has been reported since the 1980s’ [1-4]. This trend may be linked to higher maternal weight gain during pregnancy, increase in frequencies of maternal obesity and diabetes, and reduced smoking in pregnant women [5,6]. Primary concern about the birth of a macrosomic foetus is adverse neonatal outcomes including stillbirth and neonatal mortality secondary to birth asphyxia, shoulder dystocia, birth injury, metabolic disorders, and meconium aspiration syndrome. The occurrence of these unfavourable outcomes and their risks factors have been widely studied [7-11]. Similarily, maternal complications are increased in the setting of fetal macrosomia [7-9,12-14]. These complications have been studied mainly by comparing women delivering macrosomic newborns to women delivering non-macrosomic newborns, thereby using fetal birth weight as a primary risk factor. Little attention has been paid to parameters other than fetal weight that may specifically occur in women delivering macrosomic infants.

The objective of this study was to identify risks factors, other than fetal birth weight, for maternal complications in women who delivered macrosomic infants.

## Methods

All women with singleton pregnancies who attempted a vaginal birth and delivered infants weighing at least 4000 g, in two French tertiary care centers of Paris suburbs (Hôpital Antoine Béclère, Clamart and Centre Hospitalier Intercommunal de Poissy-Saint Germain, Poissy) from January 2005 through December 2008 were included. Demographic characteristics, obstetrical history, pregnancy and neonatal data were registered prospectively in hospitalization databases (approval by CNIL, the French Data Protection Authority, under the notification number 1181076). Three types of maternal complications were considered: 1) the occurrence of a third- or fourth-degree perineal laceration; 2) a severe postpartum hemorrhage defined as persistent bleeding more than 500 cc requiring the use of prostaglandins (sulprostone), uterine artery embolization, internal iliac artery ligation or haemostatic hysterectomy; 3) the need for blood transfusion. A composite criterion was then built based on the occurrence of at least one of these 3 types of maternal complications. The criterion was named “MC”, and was used as the primary outcome in this study. Apart from fetal weight, risk factors for MC were screened among demographic characteristics, obstetrical history, pregnancy and neonatal data.

Although neonatal complications were not the primary interest of this study, two of them were considered for qualitative analysis: shoulder dystocia, defined as the need to use obstetrical maneuvers to extract the body of the fetus after head delivery; and brachial plexus injury, defined as paralysis or inability to actively move of an upper extremity as determined and diagnosed by pediatricians and neonatologists.

After a descriptive study of maternal characteristics and obstetrical outcomes, we performed multiple logistic regression analysis to best fit the model for predicting our composite criterion, MC [15]. Birthweight was included as a dichotomous variable (lower or greater than 4500 g). Other variables with *p* values < 0.2 in the univariate analysis, or known risk factors of MC (such as diabetes, body mass index and gestational age at delivery) were entered into the multivariate logistic regression model [16]. A systematic adjustment was made for the given hospital centre. In case of a statistically significant odds ratio greater than 1, we computed attributable risk [17] for the corresponding risk factor. Regardless of the certainty of causal association, the calculated attributable risk serves to quantify a portion of each factor present in MC.

In the context of macrosomia, the place given to caesarean sections is questionable. On the one hand, they may prevent maternal complications (in particular perineal lacerations), on the other hand, they may be considered as a complication of delivery by themselves. We chose to exclude women with elective cesarean delivery (defined as cesarean deliveries scheduled 8 hours or more before delivery and performed as intended [18]) since they were mainly planned due to maternofetal dystocia. However, we included other cesarean deliveries since they reflected a prolonged and difficult labor that may implicate maternal complications. To validate our selection of cesarean sections, we performed two sensitivity analyses on all cesarean sections, both elective and non-elective: the first with the same composite criteria as MC, and the second including elective cesarean delivery for macrosomic suspicion as a supplementary criteria for MC.

Statistical analyses were performed using STATA® v.11 (Stata Corporation, College Station, TX, USA) software.

## Results and discussion

### Results

During the 4-year period 2005–2008, 27630 patients delivered in the two centres. Among them, 1 832 (6.6%) women had a newborn heavier than 4000 g, of which 268 (15%) had an elective cesarean section and 1564 (85%) attempted a vaginal delivery and were included in the study. The proportion of macrosomia remained steady over the 4-year study period. Most of the patients were European (71%), had a mean body mass index (BMI) before pregnancy of 23.9 (10% had a BMI ≥ 30 before pregnancy) and did not experience diabetes during or before pregnancy (92.7%) (Table 1). Mean weight gain during pregnancy was 14 kg (range: -6 kg; 42 kg). Median duration of pregnancy was 40.5 weeks (range 36.9 – 42 weeks). Labor was induced in 33%, mainly for post-term pregnancy (60%) or maternal - fetal reasons (30%) such as hypertension, diabetes or oligohydramnios. Median duration of labor was 6 hours with 34% patients delivering before 5 hours, 52% patients delivering between 5 and 9 hours, and 14% delivering after more than 10 hours. Two hundred and sixty two women (17%) experienced a cesarean delivery during labor with two main indications: non progressive labor/dystocia (65%) and non-reassuring fetal heart rate (34%). First degree perineal tears occurred in 26% of women, and 2nd, 3rd and 4th degree in 36%, 0.9% and 0.1% respectively. Two hundred and sixty one patients (17%) experienced postpartum hemorrhage with different treatment required: oxytocin (69%), prostaglandins (sulprostone) (27%), uterine artery embolization (2%), internal iliac artery ligation (2%), and haemostatic hysterectomy (0.5%). Overall, 95 patients (6%) presented maternal complications according to the MC composite criterion. Regarding neonatal complications, shoulder dystocia occurred in 5.1% of deliveries and 5 children presented with transient brachial plexus injury that completely resolved within 6 months without sequelae.

**Table 1:** Sample characteristics

	**n (%)**
**Maternal age (years)**	
Mean +/− SD [range]	30.8 +/− 4.8 [17–46]
< 30 y	696 (44.5)
≥ 30y	868 (54.5)
**Maternal race**	
Europe	1111 (73)
Northern Africa	253 (17)
Central Africa	97 (6)
Asia and India	26 (2)
America (Northern and Southern)	30 (2)
**Diabetes**	
No diabetes	1438 (93)
Diet controlled gestational diabetes	97 (6)
Gestational diabetes requiring insulin	11 (0.7)
Pre-gestational diabetes	5 (0.3)
**Route of delivery**	
**Spontaneous vaginal**	1038 (66)
**Instrumental delivery**	264 (17)
Forceps delivery	59
Vacuum extraction	205
**Cesarean during labor**	262 (17)
**Maternal complications**	
**3rd or 4th degree perineal tears**^**§**^	**15 (1)**
**Severe postpartum hemorrhage**	**78 (5)**
Sulprostone	68
Uterine artery embolization	4
Internal iliac artery ligation	5
Haemostatic hysterectomy	1
Transfusion	12 (1)
Total*	**95 (6)**
**Neonatal complications**	
**Shoulder dystocia**	80 (5)
**Birth injury**	19 (1)
Fracture**	14
Brachial plexus injury	5
**Neonatal death**	1

^**§**^ Among vaginal deliveries.

*At least one of the maternal complications (3rd or 4th degree perineal tears or severe postpartum hemorrhage or transfusion).

**Clavicle: 11; Humerus: 3.

Mean birthweight was 4207 g (95% CI [4010; 4590]). Eight percent weighed more than 4500 g and 0.4% more than 5000 g. The gender was male in 67% of newborns and the 5 minute Apgar score was greater than 7 in 98.5% of cases. Seventy three newborns (4.7%) were admitted to neonatal intensive care unit, mainly for respiratory distress (43%) or hypoglycemia (30%), and for a median duration of 2 days [range 1–10].

Factors significantly associated with MC in univariate analysis were patient’s origin, history of a macrosomic infant vaginally delivered, prolonged duration of labor, cesarean delivery during labor and neonatal birth weight.

In multivariate analysis (Table 2), the adjusted odds ratios of MC were statistically significant for Asian women, prolonged labor (>10 hours), cesarean during labor and neonatal birthweight greater than 4500 kg. Multiparous women who had already vaginally delivered a macrosomic infant had a decreased risk of MC: ORa = 0.5 [0.2; 0.9] (p = 0.03). Attributable risk associated with birth weight greater than 4500 g was 10% whereas it was 17%, 12% and 3% for cesarean delivery during labor, duration of labor and Asian origin respectively. Sensitivity analyses for the classification of elective cesarean section (see methods section) gave similar results, i.e. same significant risk factors and same magnitude for attributable risks.

**Table 2:** Risks factors of maternal complications***

	aOR [95% CI]*	**p**	Attributable risk**
*Patient’s age (years)*		0.2	-
<30	1		
≥ 30	1.4 [0.8–2.4]		
*Origins*		0.04	3%
Non Asian	1		
Asian	3.1 [1.1–9.3]		
*Composite of parity and previous vaginal delivery of a macrosomic infant*		0.04	-
Nulliparous	1		
Multiparous without previous macrosomic delivery	0.8 [0.5–1.1]	0.06	
Multiparous with previous macrosomic delivery	0.5 [0.2–0.9]	0.03	
*Maternal Diabetes*	0.5 [0.2–1.7]	0.3	-
*Obesity (body mass index > 30 kg/m*^*2*^*)*	2.2 [0.9–5.4]	0.09	-
*Labor induction*	1.5 [0.9–2.4]	0.1	-
*Duration of labor*		0.02	12%
< 10 hours	1		
≥10 hours	1.9 [1.1–3.4]		
*Cesarean during labor*	2.2 [1.3–3,9]	0.004	17%
*Birth weight (grams)*		0.004	10%
< 4500	1		
≥4500	2.7 [1.4–5.1]		
*Infant gender (Male)*	1.2 [0.7–2.0]	0.5	-

*Adjusted odds ratio and confidence interval.

Adjustment for all the variables in the Table and gestational age at delivery and hospital centre.

**Computed if OR significant and greater than 1.

***Composite criteria for maternal complications: at least one of the maternal complications (3rd or 4th degree perineal tears or severe postpartum hemorrhage or transfusion).

### Discussion

We report that the occurrence of maternal complications when attempting vaginal birth is 6% among women delivering infants with a birthweight ≥ 4000 g. The prevalence of macrosomic infants and the characteristics of our sample were very similar to those depicted in previous studies [4,5,11,19-21]. The risk factors for MC were not only fetal weight but also Asian origin, long duration of labor, and cesarean during labor. It should also be noted that multiparous women with a previous vaginal delivery of a macrosomic child had a decreased risk of MC.

It is well known that maternal and neonatal morbidity increases with birthweight and especially over 4500 g [5,9,11,12,22]. Zhang et al. [11] have shown a J-shaped birthweight-specific perinatal mortality and morbidity curves, with 2 important thresholds: 4500 g and 5000 g. Over those two thresholds, complications are dramatically increased. The 4500 g threshold was always present in our study, but to a lesser extent since the sample was limited to newborns heavier than 4000 g. In turn, this intended limitation allowed us to study other factors since it decreased the role of birthweight in MC. Moreover, the large and unselected sample (all women with a newborn heavier than 4000 g were included) provided a sufficient statistical power and results that are potentially generalizable.

Handa et al. [13] found that perineal tears risk increased 2-fold in case of macrosomia, but increased also in Asian women, Filipinas, and Indian women compared to white women with OR = 1.37, 1.63 and 2.50 respectively for anal sphincter laceration. Our findings confirmed these results. Ethnic origins may be associated with differences in body type and variations in perineal anatomy [23,24]. These particularities in body tissue may increase the risk of perineal tears and, as found in our study for Asian women, may favor maternal complications.

The potentially “protective” effect of having previously vaginally delivered a macrosomic infant has been reported by Mahony et al. [25]. Maternal perineal tissues have therefore already experienced macrosomic delivery and are more likely to experience an uneventful subsequent delivery.

Maternal diabetes as well as body mass index were not significantly associated with maternal complication in our study. However, it is well known that maternal diabetes is associated with shoulder dystocia and also perineal tears [26]. Data from the literature also support that maternal obesity is associated with increased postpartum hemorrhage [27,28] and prolonged labors [29]. This discrepancy in our study may be explained by the fact that the role of these factors in maternal complications is primarily through birth weight. Another explanation may also be that the proportion of maternal diabetes and obese patients remained consistent, albeit relatively low, in our sample (respectively 7.3% and 11.5%).

Cesarean delivery is a known risk factor of postpartum hemorrhage especially when it occurs during labor and after a long duration of labor [30,31]. Similarly, prolonged labor with the use of oxytocin is the main contributor of uterine atony that account for 79% of the cases of postpartum hemorrhage [30]. In fact, the occurrence of post-partum hemorrhage is more increased when one of those parameter is associated with macrosomia [14,32]. In our study, both parameters (cesarean and prolonged labor) appear to increase significantly the occurrence of MC, probably through their influence on post-partum hemorrhage. Those results are in agreement with the literature but quantification of their association after adjusting for birth weight has never been reported.

In addition, we computed the attributable risk as an alternative means to evaluate the role of each risk factor in maternal complications associated with fetal macrosomia , Although it can not ensure the causality of association the attributable risk quantifies the magnitude of the proportion of MC that could be avoided by removing the risk factor. Attributable risk estimation is particularly useful for factors that may be modified, but is useful to a lesser extent for unchangeable factors such as ethnic origin.

Estimation of attributable risks enabled us to precisely identify that only 10% of MC may be attributed to a birth weight greater than 4500 g, which is almost identical to the contribution of prolonged labor. Cesarean during labor appeared to be the most important factor with attributable risk equal to 17%. This could be explained by the fact it is likely responsible for the highest rates of post partum haemorrhage and blood transfusion. Finally, as the sum of attributable risk did not reach 100%, we should also mentioned that a fraction of the cases of MC were not associated with identifiable risk factors.

## Conclusion

Beyond the influence of increased fetal weight, Asian origins, prolonged labor and cesarean delivery during labor may worsen maternal outcome. Obstetricians should be aware of these these parameters that could led to maternal complications such as severe perineal tears or postpartum hemorrhage.

## Consent

Written informed consent was obtained from the patient for publication of this report and any accompanying images.

## Competing interest

The authors report no conflict of interest.

## Authors’ contributions

FF conceived the study (concept and design), performed the statistical analysis and drafted the manuscript. JB helped in the statistical analysis and to draft the manuscript. PR and MVS helped in drafting and revisions of the manuscript. All authors read and approved the final manuscript.

## Pre-publication history

The pre-publication history for this paper can be accessed here:

http://www.biomedcentral.com/1471-2393/13/90/prepub
